# An integrative multi-omics approach reveals new central nervous system pathway alterations in Alzheimer’s disease

**DOI:** 10.1186/s13195-021-00814-7

**Published:** 2021-04-01

**Authors:** Christopher Clark, Loïc Dayon, Mojgan Masoodi, Gene L. Bowman, Julius Popp

**Affiliations:** 1 Institute for Regenerative Medicine, University of Zürich, Wagistrasse 12, 8952 Schlieren, Switzerland; 2 Nestlé Institute of Health Sciences, Nestlé Research, EPFL Innovation Park, 1015 Lausanne, Switzerland; 3 Nestlé Institute of Food Safety & Analytical Sciences, Nestlé Research, EPFL Innovation Park, 1015 Lausanne, Switzerland; 4 Institut des Sciences et Ingénierie Chimiques, Ecole Polytechnique Fédérale de Lausanne, 1015 Lausanne, Switzerland; 5 Institute of Clinical Chemistry, University Hospital Bern, Bern, Switzerland; 6 Department of Neurology, NIA-Layton Aging and Alzheimer’s Disease Center, Oregon Health & Science University, Portland, USA; 7 Old Age Psychiatry, Centre Hospitalier Universitaire Vaudois, Rue du Bugnon 46, 1011 Lausanne, Switzerland; 8 Department of Geriatric Psychiatry, University Hospital of Psychiatry Zürich, Centre for Gerontopsychiatric Medicine, Minervastrasse 145, P.O. Box 341, 8032 Zürich, Switzerland

**Keywords:** Alzheimer’s disease, CSF, MOFA, Multi-omics, Biomarkers

## Abstract

**Background:**

Multiple pathophysiological processes have been described in Alzheimer’s disease (AD). Their inter-individual variations, complex interrelations, and relevance for clinical manifestation and disease progression remain poorly understood. We hypothesize that specific molecular patterns indicating both known and yet unidentified pathway alterations are associated with distinct aspects of AD pathology.

**Methods:**

We performed multi-level cerebrospinal fluid (CSF) omics in a well-characterized cohort of older adults with normal cognition, mild cognitive impairment, and mild dementia. Proteomics, metabolomics, lipidomics, one-carbon metabolism, and neuroinflammation related molecules were analyzed at single-omic level with correlation and regression approaches. Multi-omics factor analysis was used to integrate all biological levels. Identified analytes were used to construct best predictive models of the presence of AD pathology and of cognitive decline with multifactorial regression analysis. Pathway enrichment analysis identified pathway alterations in AD.

**Results:**

Multi-omics integration identified five major dimensions of heterogeneity explaining the variance within the cohort and differentially associated with AD. Further analysis exposed multiple interactions between single ‘omics modalities and distinct multi-omics molecular signatures differentially related to amyloid pathology, neuronal injury, and tau hyperphosphorylation. Enrichment pathway analysis revealed overrepresentation of the hemostasis, immune response, and extracellular matrix signaling pathways in association with AD. Finally, combinations of four molecules improved prediction of both AD (protein 14-3-3 zeta/delta, clusterin, interleukin-15, and transgelin-2) and cognitive decline (protein 14-3-3 zeta/delta, clusterin, cholesteryl ester 27:1 16:0 and monocyte chemoattractant protein-1).

**Conclusions:**

Applying an integrative multi-omics approach we report novel molecular and pathways alterations associated with AD pathology. These findings are relevant for the development of personalized diagnosis and treatment approaches in AD.

**Supplementary Information:**

The online version contains supplementary material available at 10.1186/s13195-021-00814-7.

## Background

Along with amyloid pathology and tau-related neurodegeneration, multiple other molecular alterations and pathway dysregulations have been reported in Alzheimer’s disease (AD). Indeed, there is strong evidence that pathophysiological changes involving neuroinflammation [1] lipid metabolism [2], one-carbon metabolism [3], amino acids [4], and glucose metabolism [5], among others, are present in AD. However, the contribution and relevance of these alterations to clinical manifestation and progression of the disease as well as their inter-individual variations, and complex interrelations, remain poorly understood. While these processes are generally not considered part of the “core” AD pathology, they may substantially contribute to the development of amyloid pathology and neurodegeneration and precipitate the manifestation of symptoms. As they may be occurring at early clinical and preclinical disease stages, a better understanding of these processes may be highly relevant for both early diagnosis and prognosis and the design of targeted interventions to interfere with developing AD pathology and clinical disease progression.

‘Omics approaches and technologies have made major progress over the past decade to resolve the complexity of the metabolome, lipidome and proteome [6]. As powerful phenotyping technologies, ‘omics significantly accelerate the understanding of mechanisms of pathophysiological alterations that underlie complex diseases such as AD [7, 8]. Beyond the potential of identifying altered biofluid molecule profiles that could be used as biomarkers, these technological advances also offer the opportunity to explore different types of molecules in parallel by combining multiple ‘omics methods. Recent statistical advances have made it possible to integrate the information from multiple data modalities for a thorough exploration of endophenotype networks and biological interactions related to disease [9]. While multi-omics approaches have recently shown their potential in relation to different other pathological conditions [10–12], these methods still need to be more broadly adapted and applied in AD [13].

Here, we hypothesized that specific patterns of proteins, lipids, neuroinflammatory markers, and metabolites are associated with core features of the AD pathology and indicate disease-related, inter-connected biological pathway alterations. We investigated these alterations across multiple biochemical pathways by using a multi-layer dataset acquired by analysis of cerebrospinal fluid (CSF) from a cohort of elder subjects with normal cognition. In order to integrate data from different ‘omics platforms in an unbiased fashion while considering interactions between modalities, we combined different approaches including single ‘omics analysis and multi-omics factor analysis (MOFA) [14–16].

## Methods

### Study population

One hundred and twenty community dwelling individuals, aged 55 or older, including subjects with normal cognition, mild cognitive impairment (MCI), or mild AD dementia (defined as previously described [3]), were enrolled into a brain aging study conducted in the Department of Psychiatry and the Department of Clinical Neurosciences, University Hospital of Lausanne, Switzerland. They were recruited among memory clinic outpatients or through advertisement. An overall clinical, neurological, and comprehensive neuropsychological assessment was performed between 2013 and 2016, which included the Mini Mental State Examination (MMSE, [17]) and Clinical Dementia Rating (CDR, [18]). Candidates with unstable medical conditions or with neurological or psychiatric diseases that could interfere with cognitive performance were excluded as previously described [19]. Clinical and neuropsychological follow-up evaluations were performed at 18 and 36 months using the same methods and tests.

### Study procedures

#### Clinical assessment

We determined Mini-Mental State Examination (MMSE), CDR, and CDR sum of boxes (CDR-SoB), for all participants. CDR-SoB and CDR were based on the information available from the participant and his/her relative, the clinical examination, and comprehensive neuropsychological test performance, as previously described [19].

#### Biochemical sample collection and handling

Ten to 12 ml of cerebrospinal fluid (CSF) obtained from lumbar punctures conducted after an overnight fast at participant inclusion were spun down at 4 °C, immediately aliquoted, and snap frozen at − 80 °C until assayed [19], with no freeze-thaw cycles allowed. Samples were stored for a maximum of 3 years before analysis. Study personnel blinded to clinical data performed biochemical and genetic analyses.

#### Cerebrospinal fluid AD biomarkers

CSF beta-amyloid 1-42 (Aβ_1-42_), total-tau (Tau), and tau phosphorylated at threonine 181 (P-Tau) concentrations were measured using commercially available ELISA kits (Fujirebio, Gent, Belgium) in all samples within the cohort.

#### Analyte measurements

Multiple ‘omics data from different pathways and various biological levels were acquired from a vast majority of participants within the cohort (*n* = 114/120 for proteomics, 118 for metabolomics, 119 for neuroinflammation and one-carbon metabolism, and 120 for lipidomics).

CSF samples were measured using an untargeted shotgun proteomic workflow based on liquid chromatography (LC) tandem MS (MS/MS) using an Ultimate 3000 RSLC nano system and a hybrid linear ion trap-Orbitrap (LTQ-OT) Elite (Thermo Scientific, San Jose, CA, USA) [20, 21]. Relative quantification of proteins between the samples was obtained using isobaric tagging with the tandem mass tag technology [22]. Full experimental details and parameters of the proteomic analysis have been published previously [23, 24]. A targeted subset of thirty-seven CSF inflammatory proteins including IFNγ, IL-1β, IL-2, IL-4, IL-6, IL-8, IL-10, IL-13, TNFα, IL-1α, IL-5, IL-7, IL-12/23p40, IL-15, IL-16, IL-17A, TNFβ, VEGFA, Eotaxin, MIP-1β, Eotaxin-3, TARC, IP-10, MIP-1α, MCP-1, MDC, MCP-4, VEGF-C, VEGF-D, Tie-2, sFLT-1 (VEGFR-1), PIGF-1R, bFGF, SAA1, CRP, sVCAM-1, and sICAM-1 were separately quantified using a sandwich immunoassay (Meso Scale Diagnostics (MSD), Rockville, MD, USA), according to the manufacturer’s instructions. This platform has been validated by the manufacturer (https://www.mesoscale.com/~/media/files/product%20inserts/neuroinflammation%20panel%201%20human%20insert.pdf) and has been previously used successfully [25].

CSF lipids were quantified using an MS-based shotgun approach [26]. This technology can cover 22 quantifiable different lipid classes encompassing more than 200 lipid species; it achieves absolute quantification, by inclusion of internal standards for every lipid class measured. Figure-of-merits are an average coefficient of variation of < 10% (intra-day), approximatively 10% (inter-day), and approximatively 15% (inter-site) for most lipid species.

Metabolomic profiling was carried out by means of ^1^H NMR spectroscopy, as reported previously [27]. This approach covered major metabolic pathways, including amino acids, carboxylic acids, and central energy metabolism. Metabolites within the one-carbon pathway are a hypothesis-driven subset of metabolites [3] and were separately analyzed using LC-MS/MS as previously described [28] with an Accela UHPLC 1250 Pump coupled to a TSQ Quantum Vantage triple quadrupole mass spectrometer equipped with a heated electrospray ionization source (Thermo Scientific). Selected reaction monitoring transitions have been described previously [28].

The initial number of analytes measured in CSF, the final number of analytes selected per platform (a total of 891 analytes covered), and quantification method used for each platform are summarized in Table 1.

**Table 1:** Datasets used in this study

Dataset	Analytes initial/final	Quantification technique	References
Proteomics	791/768	LC-MS/MS	[23, 24]
Neuroinflammation	38/21	Multi-array sandwich immunoassay	[29–31]
One-carbon metabolism	17/9	LC-MS/MS	[28, 32]
Metabolomics	71/63	^1^H NMR	[27]
Lipidomics	65/26	MS	[26, 33]
Biomarkers of core AD pathology	3/3	ELISA	[30]

Available datasets from the cohort along with the number of analytes measured in this study and the associated quantification methods. For each dataset the initial number of analytes quantified, the number of measurements remaining after quality control, quantification technique used, and technical references are indicated. LC-MS/MS, liquid chromatography tandem mass spectrometry; ^1^H NMR, proton nuclear magnetic resonance; MS, mass spectrometry; ELISA, enzyme-linked immunosorbent assay

#### Genetic measures

The *APOE* genotype was determined by PCR as previously described [29]. Participants with one or more e4 alleles were classified as carriers.

### Data preparation and transformation

#### Lipidomics

Twenty-six high-quality intact lipids with less than 5% of null values were selected as continuous numerical markers from 65 original measurements. Numerical lipid marker values were log10-transformed prior to analysis.

#### Metabolomics

Seventy-one peak integrals were originally measured in CSF. Sixty-three analytes with less than 5% missing values were selected from the obtained spectra. Peak integral values were log10-transformed prior to analysis.

#### One-carbon metabolomics

Seventeen analytes were initially measured in CSF. Some analytes could not be measured in the majority of samples and were excluded from the analysis (i.e., homocysteic acid, dimethylglycine, betaine, total homocysteine, pyridoxine, and pyridoxamine); taurine and glycine data were inconsistent and were also filtered out resulting in 9 measured analytes (i.e., choline, cystathionine, methionine, riboflavin, S-adenosylhomocysteine, S-adenosylmethionine, serine, cysteine and 5-methyltetrahydrofolate). Analytes with more than 5% missing data points were also removed. Numerical values were log10-transformed prior to analysis.

#### Neuroinflammatory markers

Thirty-eight markers were measured in CSF. Calibration curves, batch effects, and analytes with more than 5% missing data points were removed, and lower limit of quantification was controlled. After this quality control, 17 markers were removed, resulting in 21 markers selected. Concentrations were log10-transformed prior to analysis.

#### Proteomics

Relative quantification data were available for all subject samples as log2 ratios as previously described [24]. Analytes with more than 5% missing data points were removed, resulting in 768 proteins measured from an initial number of 791.

Before analysis, outliers (i.e.*,* data points that exceeded the cutoff value of mean ± 3 × standard deviation) were replaced by the cutoff value in all datasets (*n* = 28 for lipidomics, 36 for metabolomics, none for one-carbon metabolomics, 17 for neuroinflammatory markers, and 345 for proteomics). For all datasets, this represented below 1% of all data points. Missing values were replaced using an iterative Markov chain Monte Carlo method before single-modality feature selection approaches, but were not replaced for multi-omics analysis as the MOFA method can handle large proportions of missing values [14].

### Statistical and analytical approaches

Descriptive statistics for the cohort were performed using *t* tests comparing AD and control groups for continuous variables and chi-square tests for categorical variables. Data was clustered by hierarchical clustering across samples and factors values or loadings.

#### Feature selection methods

##### Single-modality approaches

To overcome the bias resulting from correlation between variables and thus unreliability and saturation of standard regression techniques, we used Elastic-Net regularization (*α* = 0.5) for regression analysis. This was performed separately for each individual ‘omics platform in the whole cohort using R software with custom routines implementing the *glmnet* package [34]. Each pre-specified CSF biomarker endpoint was considered as a continuous dependent variable and associated features were identified using a value of λ (lambda) that minimized the 10-fold cross validated error.

##### Multi-omics factor analysis

This analysis was performed using the MOFA package on the whole cohort in R and Python software [14]. Latent factors (also referred as LFs) were selected to explain a minimum of 2% variance in at least one data type. The MOFA model was trained over 938 iterations with a convergence threshold of 0.1. Individual analytes were selected if their normalized absolute loading value was > 0.8 within any given LF in order to include only analytes with strong associations. More details about the MOFA method can be found in Additional file 1. The trained MOFA model was validated using both a correlation approach and CSF AD biomarker predictions (Additional file 2, Figures S1 and S2, respectively).

##### Associations with CSF biomarkers of AD

In order to evaluate the correlations of the analytes identified by the MOFA model with CSF biomarkers of AD (CSF Aβ_1-42_, Tau and P-Tau), we used two-tailed Spearman’s rho. Benjamini-Hochberg correction of *P* value for multiple testing was then applied using a false-discovery rate of 0.1.

##### Models for the prediction of AD and of cognitive decline

Predictions were ran using the *glm* package in R. Subjects were classified as controls or AD, according to the presence or absence of an AD CSF biomarker profile, defined by a CSF P-Tau/Aβ_1-42_ ratio > 0.0779 based on center data [30]. We constructed a reference model including the following covariates for AD prediction: age, sex, years of education, baseline MMSE score, and APOE4 carrier status. MMSE change at last available follow-up for one hundred and three participants, with nineteen participants followed up at 18 months only, and eighty-four at 36 months, was used to classify participants with decreased global cognition as follows: MMSE score at baseline – MMSE at last follow-up visit ≥ 2. Another reference model including age, sex, years of education, baseline MMSE score, APOE4 carrier status, and time to last follow-up was constructed for cognitive decline prediction. We then used an iterative approach, first adding all analytes identified by the MOFA model individually to the above reference model and selected the model displaying the smallest Akaike information criterion (AIC) value to select the best molecule to add at each iteration. We repeated this process over successive iterations, adding a single analyte each time. Performance of the models was analyzed by comparing area under the curve (AUC) of the resulting ROC curves using the DeLong method. No further improvements to the AUC were observed after five iterations for both predictions. Confusion matrices to assess sensitivity and specificity were calculated for all models.

#### Pathway enrichment

Proteins selected by the MOFA model were searched for in the UniProt database [35], and their entry number was then subsequently used within the Reactome database [36]. A separate over-representation analysis was performed for each LF. This analysis used hypergeometric distribution to determine which pathways and biological reactions were over-represented within the dataset. Over-represented pathways were then manually grouped into broader ontology-based categories (Additional file 3, Table S1).

## Results

### Cohort description

The clinical and demographical characteristics of the participants included in this study are shown in Table 2.

**Table 2:** Study cohort

	Whole cohort (***n*** = 120)	Control (***n*** = 79)	AD (***n*** = 41)	***P*** value
Age (years)	70.37 ± 7.92	68.42 ± 8.23	74.15 ± 5.7	< 0.001
Sex (%, female)	64.2	67.1	58.5	0.354
Education (years)	12.37 ± 2.7	12.51 ± 2.7	12.10 ± 12.1	0.404
CDR-SoB	1.054 ± 1.6	0.456 ± 0.9	2.20 ± 2.0	< 0.001
MMSE	26.94 ± 3.08	27.85 ± 2.28	25.15 ± 3.71	< 0.001
P-Tau/Aβ_1-42_ ratio	0.088 ± 0.082	0.048 ± 0.127	0.165 ± 0.104	< 0.001
APOEε4 carrier (%)	29.6	17.7	56.1	< 0.001

Characteristics of the study cohort. Mean values ± standard deviation are presented. Per definition, participants within the AD group all presented a positive AD CSF biomarker profile, defined by a P-tau/Aβ_1-42_ ratio > 0.0779. *P* value was obtained from *t* test for continuous variables or chi-square statistics for sex

### Single-modality feature selection

**Fig. 1:**
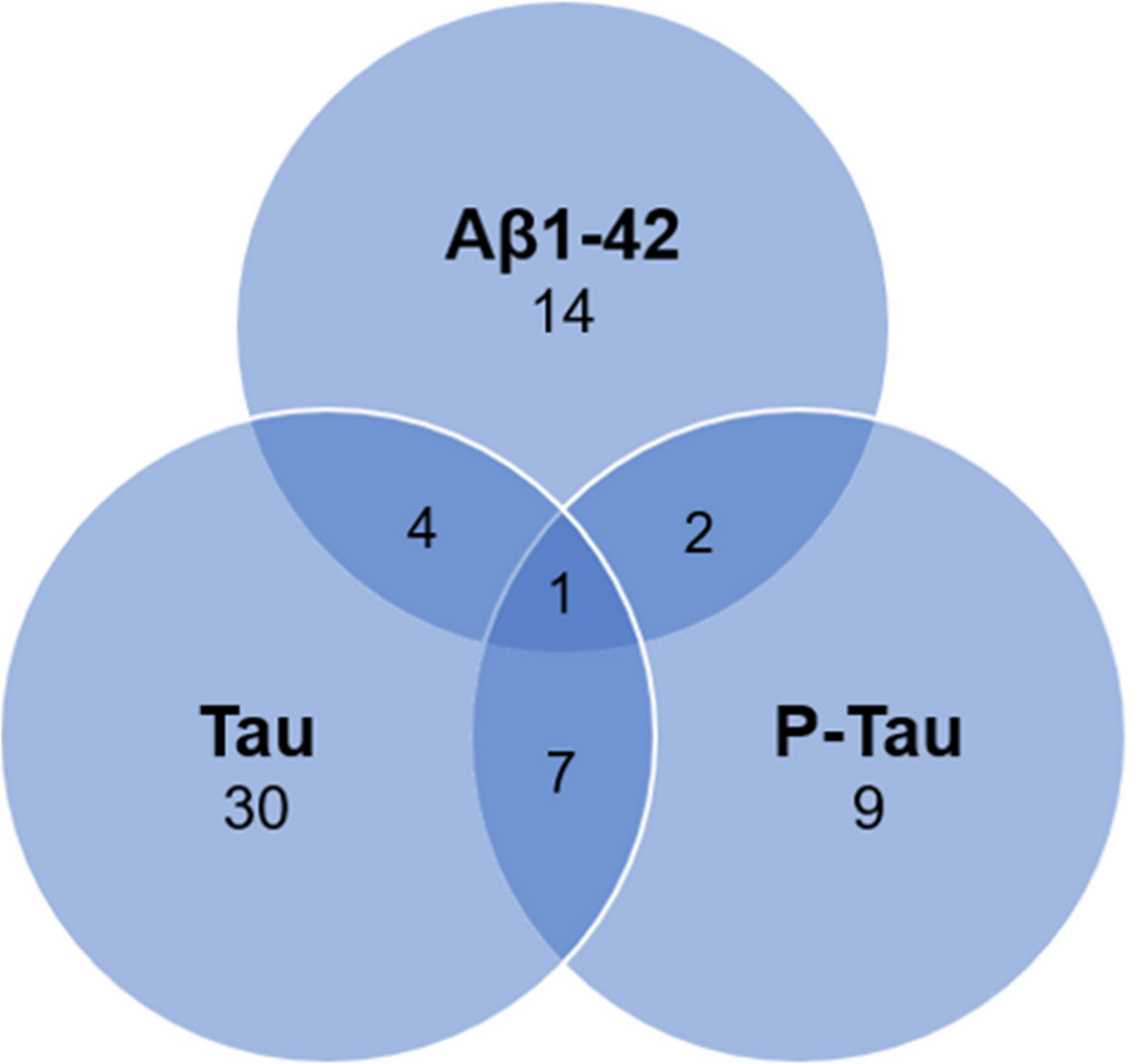
Venn diagram of associations with CSF core AD biomarkers. Venn diagram of associations of analytes obtained by regression models with CSF core AD biomarkers. Number of molecules identified as well as those shared between biomarkers is shown. The full list of associated molecules is presented in Tables 3, 4, and 5

Elastic-Net regression within each single ‘omics modality identified 82 molecules associated with CSF “core” biomarkers of AD pathology (i.e., Aβ_1-42_, Tau and P-Tau) within the whole cohort (Tables 3, 4, and 5). Twenty out of the thirty-seven proteins selected were correlated with at least one CSF AD biomarker. Only two neuroinflammatory molecules displayed no correlation with CSF AD biomarkers. Conversely, only two molecules at metabolomics level and two lipids were correlated with CSF AD biomarkers. Finally, total cysteine showed no correlations (Additional file 3, Table S2). Strikingly, distinct panels of CSF analytes were associated with either Aβ_1-42_, or Tau and P-Tau, reflecting alterations of different pathways in relation to amyloid pathology, neurodegeneration, and tau pathology, with very little overlap (Fig. 1). Only protein 14-3-3 zeta/delta was associated with all three biomarkers.

**Table 3:** Analytes associated with CSF Aβ_1-42_

	Coeff.
**Neuroinflammation**	
C-reactive protein	17.9542
Monocyte chemoattractant protein-1	− 78.6254
**Proteomics**	
Spermine synthase	109.3853
WAP four-disulfide core domain protein 2	93.78915
Ephrin-B2	72.52614
Neuroendocrine convertase 2	55.25737
WAP four-disulfide core domain protein 1	42.96994
Spectrin beta chain, non-erythrocytic 5	37.99513
Neuropentraxin2	37.29243
Chondroadherin	32.78591
Reelin	19.927
Sodium/potassium-transporting ATPase subunit alpha-2	17.62631
von Willebrand factor	17.59776
Mast/stem cell growth factor receptor kit	17.49956
Lymphatic vessel endothelial hyaluronic acid receptor 1	7.896977
Neurotrimin	1.179857
Acid ceramidase	0.892224
Protein shisa-6	0.412964
Monocyte chemoattractant protein 1	−12.5945
SPARC related modular calcium binding 1	− 186.61
14-3-3 protein zeta/delta	− 230.535

Analytes within the whole cohort with a significant association with CSF Aβ_1-42_ sorted by decreasing absolute association strength within each modality. For each analyte, the coefficient obtained by Elastic-Net regression is shown

**Table 4:** Analytes associated with CSF Tau

	Coeff.
**Lipidomics**	
DAG 34:0	1080.894
PC 32:0	482.8502
PC 34:1	357.532
LPA 16:0	117.3799
SE 27:1 18:3	− 15.4237
SE 27:1 18:2	− 22.0295
**Metabolomics**	
Glycoproteins	519.5138
3-Hydroxyisovaleric acid	154.6212
Hydroxybutyric acid	71.2998
S56	− 5.1297
S62	− 49.1323
Glucose	− 134.352
**One-carbon metabolism**	
S-adenosylhomocysteine	264.4874
Choline	15.9208
5-Methyltetrahydrofolate	− 361.534
**Neuroinflammation**	
Soluble fms-like tyrosine kinase 1	473.9178
Il-15	173.1296
Soluble vascular cell adhesion molecule-1	159.6389
Soluble intracellular cell adhesion molecule-1	90.9487
Monocyte chemoattractant protein-1	41.9127
**Proteomics**	
14-3-3 protein zeta/delta	283.67
brain abundant membrane attached signal protein 1	107.72
SPARC related modular calcium binding protein 1	64.03
Neuromodulin	55.50
Fructose-biphosphate aldolase A	52.77
Neurofilament medium polypeptide	49.02
Transgelin-3	31.42
Secreted-frizzled-related protein 4	− 1.86
Chondroadherin	− 7.99
Dynein heavy chain 10, axonemal	− 11.34
Glia-derived nexin	− 13.09
A-kinase anchor protein 11	− 18.52
Reelin	− 18.86
Augurin	− 43.98
Spectrin beta chain, non-erythrocytic 1	− 52.34
Sialate O-acetylesterase	− 56.71
Proline-rich acidic protein 1	− 80.54
Fibromodulin	− 84.41
Cathepsin D	− 144.10
Insulin-like growth factor-binding protein 7	− 169.27
Ectonucleotide pyrophosphatase/phosphodiesterase family member 2	− 213.32

Analytes within the whole cohort with a significant association with CSF Tau sorted by decreasing absolute association strength within each modality. For each analyte, the coefficient obtained by Elastic-Net regression is shown. S56 and S62 represent different unidentified metabolites

**Table 5:** Analytes associated with CSF P-Tau

	Coeff.
**Lipidomics**	
PC 34:1	75.7244
PC 32:0	41.1825
DAG 34:0	32.4248
SE 27:1 18:1	9.313
LPE 22:6	1.2951
SE 27:1 18:2	− 13.6849
**Metabolomics**	
Glycoproteins	42.4426
3-Hydroxyisovaleric acid	15.143
S61	− 0.3484
S59	− 1.5514
S56	− 15.8893
**One-carbon metabolism**	
S-adenosylhomocysteine	48.6705
5-Methyltetrahydrofolate	− 72.7586
**Proteomics**	
SPARC-related modular calcium binding 1	4.388161
Brain abundant membrane attached signal protein 1	4.028155
Neuromodulin	1.626098
Thymosin beta-10	1.291504
14-3-3 protein zeta/delta	1.10139
Pyruvate kinase PKM	0.189499

Analytes within the whole cohort with a significant association with CSF P-Tau sorted by decreasing absolute association strength within each modality. For each analyte, the coefficient obtained by Elastic-Net regression is shown. S56, S59 and S61 represent different unidentified metabolites

### Overview of the MOFA model

**Fig. 2:**
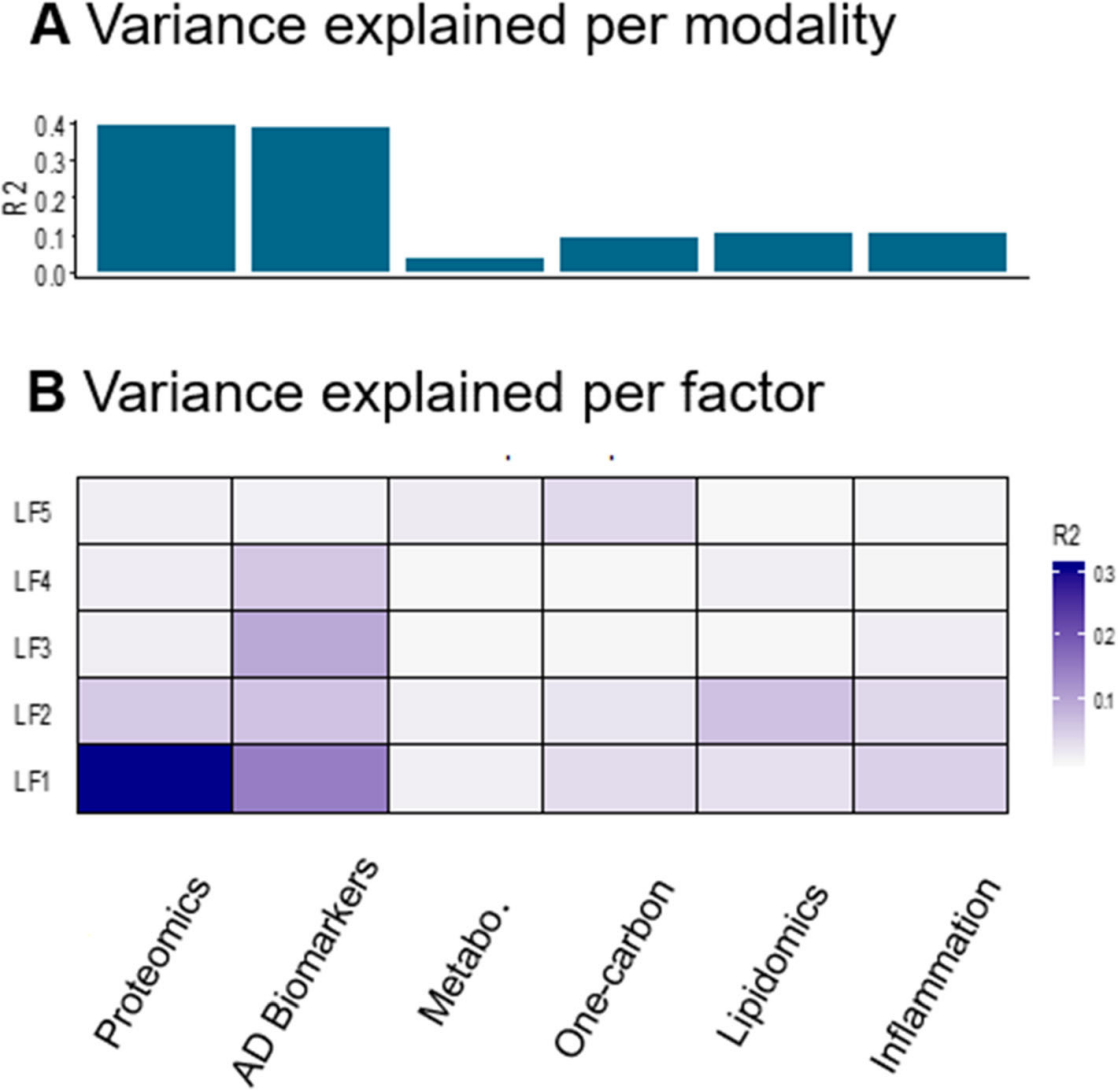
Overview of the MOFA model. Overview of the trained MOFA model showing variance (R2) within the cohort explained by each modality (top) and latent factors (LFs, bottom) from the trained MOFA model

**Fig. 3:**
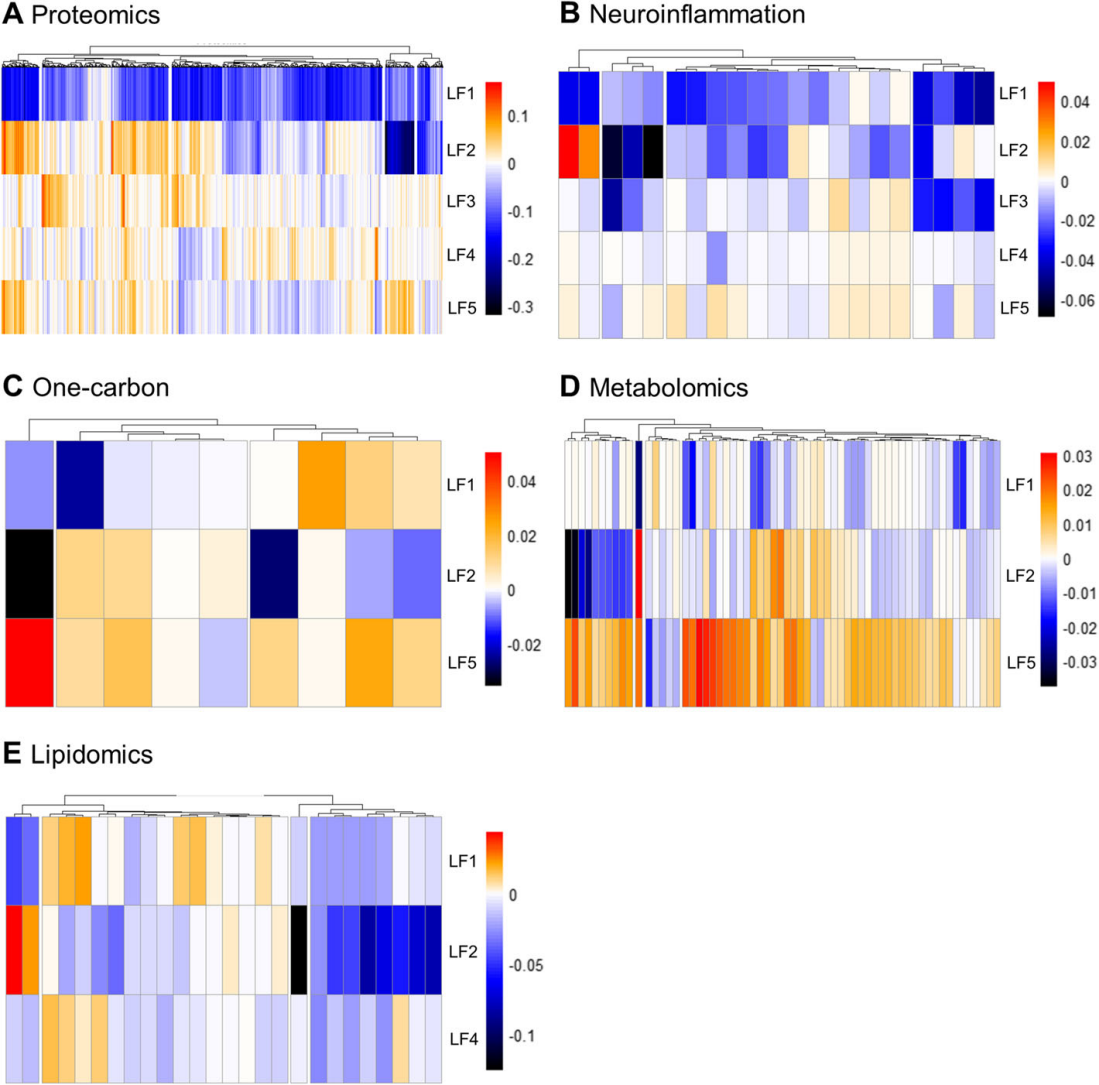
Clustering of loadings across latent factors. Heatmaps of hierarchical clustering of the measured loadings across in LFs for data obtained from proteomics (**a**), neuroinflammation markers (**b**), one-carbon metabolism (**c**), metabolomics (**d**), and lipidomics (**e**) showing clusters of analytes along the *X*-axis and the association of each individual analyte with each LF (shown on the *Y*-axis). Note the distinct pattern within each LF. Color scale indicates both the direction and strength of relative associations

**Fig. 4:**
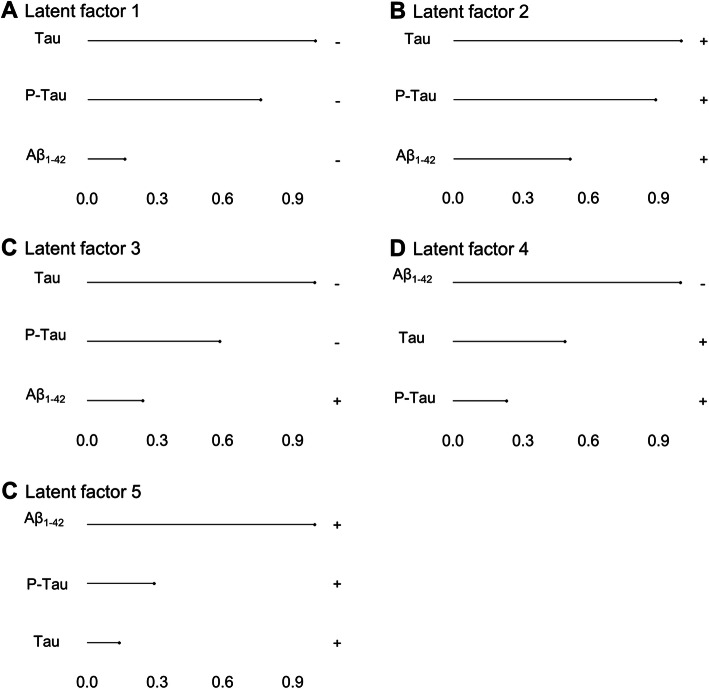
Loadings of CSF AD biomarkers. Normalized loadings of CSF AD biomarkers shown on the *X*-axis across the five latent factors of the trained MOFA model. Positive or negative signs indicate the relative direction of the CSF AD biomarkers with the associated latent factor. Note that signs are relative within a single latent factor for biomarker weights

In parallel, we trained a MOFA model on the whole cohort, to identify major dimensions of heterogeneity (latent factors; LFs) responsible for the variance within the cohort. This model identified five LFs that each explained a minimum of 2% variance in at least one of the analyzed metabolic levels. Among these factors, LF1 and LF2 were present in most multi-omics modalities, revealing a broad participation to variance within the cohort (Fig. 2). On the other hand, the remaining LFs only captured variance across some modalities (three for LF4 and LF5, two for LF3) and had a smaller contribution to overall variance. Across all LFs, the CSF AD biomarkers accounted for 38.5%, proteins 39.8%, lipids 10.3%, neuroinflammation markers 10.3%, one-carbon metabolites 9%, and other metabolites 3.7% of the variance contained within the cohort (Fig. 2). We next produced clustered heatmaps of the weight (i.e., the association of an individual molecule with the LF) of each analyte across different LFs (Fig. 3a–e) revealing specific patterns of associations between analytes within each analyzed multi-omic level and LFs. For example, a subset of proteins with a negative association with LF1 have a positive association with LF2 (Fig. 3a) and molecules within the one-carbon metabolism are differentially associated with LF1 and LF2 (Fig. 3c). These patterns suggest groups of molecules interacting together with specific effects on LFs through common pathways. Because only three CSF core AD biomarkers were measured, we did not produce heatmaps to analyze the association of Aβ_1-42_, Tau and P-Tau with these five LFs, but rather, we inspected their absolute individual loadings across all LFs (Fig. 4). This revealed that individual CSF AD biomarkers had different contributions across the identified LFs. CSF Tau and P-Tau levels were strongly associated with LF1, LF2, and LF3, while Aβ_1-42_ was the main contributor to variance among the CSF AD biomarkers to LF4 and LF5 indicating that these latter LFs were associated with amyloid pathology and the former with tau pathology and neurodegeneration.

### Individual analyte contributions to LFs

We next addressed the contribution of individual molecules to variance within the cohort and how these molecules aligned with CSF AD biomarkers. We selected analytes with absolute normalized loadings > 0.8 within any given LF derived from the MOFA model. This approach selected 37 proteins, 7 neuroinflammatory markers, 3 one-carbon metabolites, 5 lipids, and 7 other metabolites (not counting analytes selected in multiple LFs) that contributed the most to variance within the cohort (Table 6). We next investigated the relationship between the identified analytes and the expression levels of CSF AD biomarkers in the relevant LFs. We have shown that the LFs are differentially associated with the individual CSF AD biomarkers. Molecules selected within each LF are therefore associated with the CSF AD biomarkers. Indeed, twenty out of thirty-seven proteins were correlated with at least one CSF AD biomarker. Only two neuroinflammatory molecules displayed no correlation with CSF AD biomarkers. Conversely, only two molecules at metabolomics level and two lipids were correlated with CSF AD biomarkers. Finally, in one-carbon metabolism, total cysteine showed no correlations (Additional file 3, Table S2). As they were selected by the MOFA model, these molecules are part of LFs who have an effect on the variance within the cohort. By considering which LF they were selected from, we can infer that they are part of an interacting set of analytes who is associated with changes in CSF AD biomarkers. For example, despite showing no correlations with any CSF AD biomarker, total cysteine is selected by our MOFA model in LF1, LF2, and LF5. Because these LFs have strong associations with Tau, P-Tau, and Aβ_1-42_ (Fig. 4), total cysteine is related to a pathway or group of interacting molecules in these LFs that together are associated with the markers of core AD pathology. Kininogen-1 (KNG1) also displayed no correlation with CSF AD biomarker, but we can infer it is part of a group of molecules associated with Tau and P-Tau, since it was selected in LF2. In some cases, the MOFA model revealed supplementary associations. For example, cholesteryl ester SE 27:1 16:0 showed no correlations with Aβ_1-42_ and strong correlations with both Tau and P-Tau. This lipid was selected by our MOFA model in LF4, however, suggesting it is part of an interactome with a strong association with Aβ_1-42_.

**Table 6:** Analytes associated with latent factors

LF	Analyte	Full name	Entry#	Previously reported AD association
***Proteomics***				
1	NRN1	Neuritin isoform 1 precursor	Q9NPD7	
1	SMS	Spermine synthase	P52788	Yes [37]
1	NXPH4	Neurexophilin-4	O95158	
1	LTBP1	Latent-transforming growth factor beta-binding protein 1	Q14766	
1	CLUS	Clusterin	P10909	Yes [38]
1	NPDC1	Neural proliferation differentiation and control protein 1	Q9NQX5	
1	PNOC	Prepronociceptin	Q13519	
1	DYL2	Dynein light chain 2, cytoplasmic	Q96FJ2	
1	PDGFB	Platelet-derived growth factor subunit B	P01127	Yes [39]
1	SAP3	Sphingolipid activator protein 3	P17900	
1	MT1E	Metallothionein-1E	P04732	Yes [40]
1	PCSK1	Neuroendocrine convertase 1	P29120	Yes [41]
1	TAGL	Transgelin-2	P37802	Yes [42]
1	MT3	Metallothionein-3	P25713	Yes [40]
1	LY6H	Lymphocyte antigen 6H	O94772	
2	SAMP	Spindle-associated membrane protein 1	Q5SNT2	
2	VTNC	Vitronectin	P04004	Yes [43]
2	KNG1	Kininogen-1	P01042	
2	FETUA	Alpha-2-HS-glycoprotein	P02765	Yes [44]
2	HELZ	Probable helicase with zinc finger domain	P42694	
2	PLMN	Plasminogen	P00747	Yes [45]
2	PGRP2	N-acetylmuramoyl-L-alanine amidase	Q96PD5	
2	AFAM	Afamin	P43652	Yes [44]
2	ITIH1	Inter-alpha-trypsin inhibitor heavy chain H1	P19827	Yes [46]
2	CO8B	Complement component C8 beta chain	P07358	Yes [47]
2	FIBA	Fibrinogen alpha chain	P02671	Yes [48]
2	CO6	Complement component C6	P13671	Yes [47]
2	ITIH4	Inter-alpha-trypsin inhibitor heavy chain H4	Q14624	Yes [46]
3	EPDR1	Mammalian ependymin-related protein 1	Q9UM22	
3	SIAE	Sialate O-acetylesterase	Q9HAT2	
4	X1433Z	14-3-3 protein zeta/delta	P63104	Yes [49]
4	S10A6	Protein S100-A6	P06703	Yes [50]
4	PRDX6	Peroxiredoxin-6	P30041	Yes [51]
5	VTM2A	V-set and transmembrane domain-containing protein 2A	Q8TAG5	
5	S10A6	Protein S100-A6	P06703	Yes [50]
5	CMGA	Chromogranin-A	P10645	Yes [52]
5	ZP2	Zona pellucida sperm-binding protein 2	Q05996	
5	SLIK1	SLIT and NTRK-like protein 1	Q96PX8	Yes [53]
***Neuroinflammation***				
1	sVCAM-1	Circulating vascular cell adhesion molecule-1	P19320	Yes [54]
1	IL-15	Interleukin-15	P40933	Yes [54]
1	sICAM-1	Soluble intracellular adhesion molecule-1	P05362	Yes [54]
2	SAA	Serum amyloid A	P0DJI8	Yes [55]
2	PIGF_1R	Insulin-like growth factor 1 receptor	P08069	Yes [56]
3	PIGF_1R	Insulin-like growth factor 1 receptor	P08069	Yes [56]
4	IL-16	Interleukin-16	Q14005	Yes [57]
5	MCP-1	Monocyte chemoattractant protein-1	P13500	Yes [54]
5	PIGF_1R	Insulin-like growth factor 1 receptor	P08069	Yes [56]
***One-carbon metabolism***				
1	MTHF	5-methyltetrahydrofolate	20,612	Yes [58]
1	SAH	S-adenosyl-L-homocysteine	16,680	Yes [59]
2	CYST	Total cysteine	15,356	Yes [60]
3	SAH	S-adenosyl-L-homocysteine	16,680	Yes [59]
4	CYST	Total cysteine	15,356	Yes [60]
5	CYST	Total cysteine	15,356	Yes [60]
***Metabolomics***				
1	N/A	Glycoproteins	17,089	Yes [61]
2	N/A	Alanine	16,449	Yes [62]
2	N/A	Valine	27,266	
2	N/A	Glycoproteins	17,089	Yes [61]
3	N/A	Inositol	24,848	
4	N/A	Glycoproteins	17,089	Yes [61]
5	N/A	Formic acid	30,751	
5	S69	Unidentified metabolite	N/A	N/A
5	N/A	Acetoacetic acid	15,344	Yes [63]
***Lipidomics***				
1	PC 32:0	1,2-Dihexadecanoyl-sn-glycero-3-phosphocholine	N/A	Yes [64]
2	SE 27:1 18:2	Cholesteryl ester	N/A	
3	PC 32:0	1,2-Dihexadecanoyl-sn-glycero-3-phosphocholine	N/A	Yes [64]
4	SE 27:1 18:2	Cholesteryl ester	N/A	
4	SE 27:1 20:4	Cholesteryl ester	N/A	
4	SE 27:1 16:0	Cholesteryl ester	N/A	
5	LPG 20:1	1-(11Z-eicosenoyl)-glycero-3-phospho-(1′-sn-glycerol)	N/A	

CSF biomolecules significantly associated with the LFs within the MOFA model and whether they have been previously associated with AD. Entry# denotes the analyte identifier within the UniProt database (for proteomics and neuroinflammation) or ChEBI database (for other analytes)

### Cross-modality interactions

Some of the identified LFs only contain a subset of the tested modalities (Fig. 2). For instance, one-carbon metabolism and metabolomics were only weakly associated with LF3 and LF4, whereas lipidomics was nearly absent from LF3 and LF5. Therefore, the contribution of individual LFs to total variance results from a specific combination of the different ‘omics modalities. In addition, individual molecules also presented different patterns of association across LFs. For example, a subset of lipids, including PC 32:0, PC 34:1, LPA 18:3, and TAG 54:3, had a strong positive association with LF2 and a weak negative association with LF4. Since LF2 was associated with all tested modalities (Fig. 2), this indicates these analytes interact within multiple biological pathways and could be within a hub of metabolic changes. Furthermore, LF2 is associated with both Tau and P-Tau indicating neurodegeneration and tau pathology could therefore relate to a more general metabolic alteration. This is supported by the association of PC 32:0 with tau pathology in single ‘omics. In contrast, LF4 is strongly associated with amyloid pathology and it is only associated with changes in lipids and proteins (in addition to CSF AD biomarkers). Therefore, only a subset of lipids directly interacts with amyloid pathology. Taken together, these results show that the different aspects of AD pathology derive from fundamentally different biological pathways and alterations.

### Prediction of AD pathology and cognitive decline using MOFA-selected molecules

**Fig. 5:**
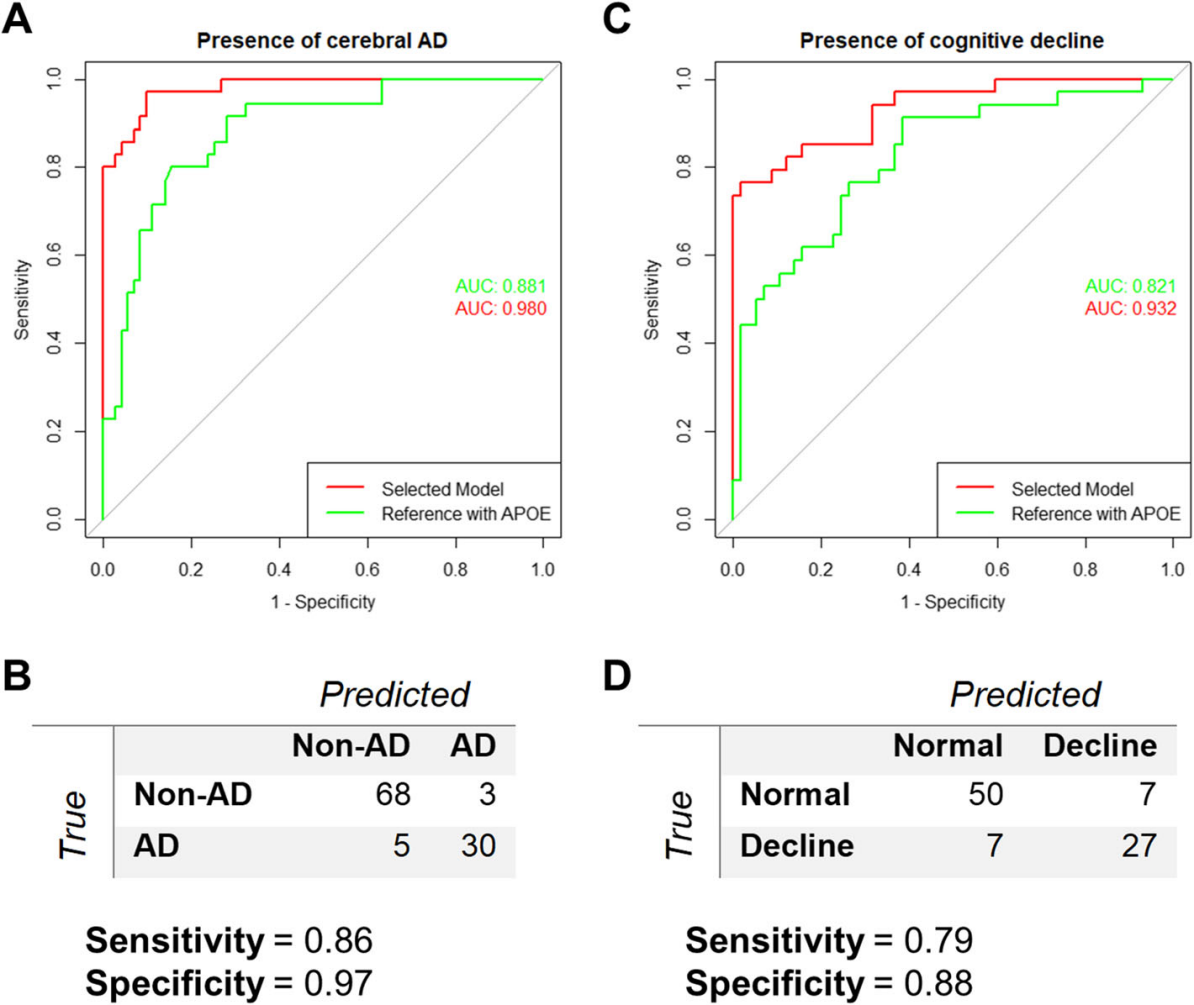
Clinical predictions. Binary logistic regression models to improve clinical predictions. **a** ROC curves and AUCs for the reference model including APOE status (green) and the final prediction model of AD pathology (red) obtained after addition of four analytes (14-3-3 zeta/delta, clusterin, interleukin-15, and transgelin-2) selected by the MOFA model. **b** Confusion matrix of the final prediction model of AD. **c** ROC curves and AUCs for the reference model including APOE status (green) and the final prediction model of cognitive decline (red) obtained after addition of four analytes (14-3-3 zeta/delta, clusterin, cholesteryl ester 27:1 16:0 and monocyte chemoattractant protein-1) selected by the MOFA model. **d** Confusion matrix of the final prediction model of cognitive decline

In addition to associations with CSF biomarkers of AD pathology, we found associations with AD reported in the literature for 37 out of 58 metabolites selected by our MOFA model (Table 6). Also, 29 of the selected proteins and 5 lipids correlated with either baseline CDR-SoB score or MMSE score Additional file 3, Table S3), while,14 of the selected proteins were associated with the presence of cognitive impairment at baseline in regression models (Additional file 3, Table S4). Therefore, in order to confirm a posteriori the importance of the molecules with high weights selected from LFs within the MOFA model, we evaluated their ability to predict either cerebral AD pathology or global cognitive decline. The model for AD prediction selected four analytes: protein 14-3-3 zeta/delta, clusterin, interleukin-15, and transgelin-2, that together improved the AUC of the ROC curve when compared to the reference model (Fig. 5a, *p* value = 0.002). In addition, both sensitivity (0.71 to 0.86) and specificity (0.87 to 0.96) were improved from the reference model. Further, adding to a reference model for the prediction of cognitive decline, four selected molecules, protein 14-3-3 zeta/delta, clusterin, cholesteryl ester 27:1 16:0, and monocyte chemoattractant protein-1, improved its AUC (Fig. 5b, *p* value = 0.0047). This also improved sensitivity (0.56 to 0.80) but not specificity (0.89 to 0.88). For both prediction of cerebral AD and of cognitive decline, the addition of single molecules to the reference models did not improve prediction (data not shown).

### Metabolic pathway enrichment

**Fig. 6:**
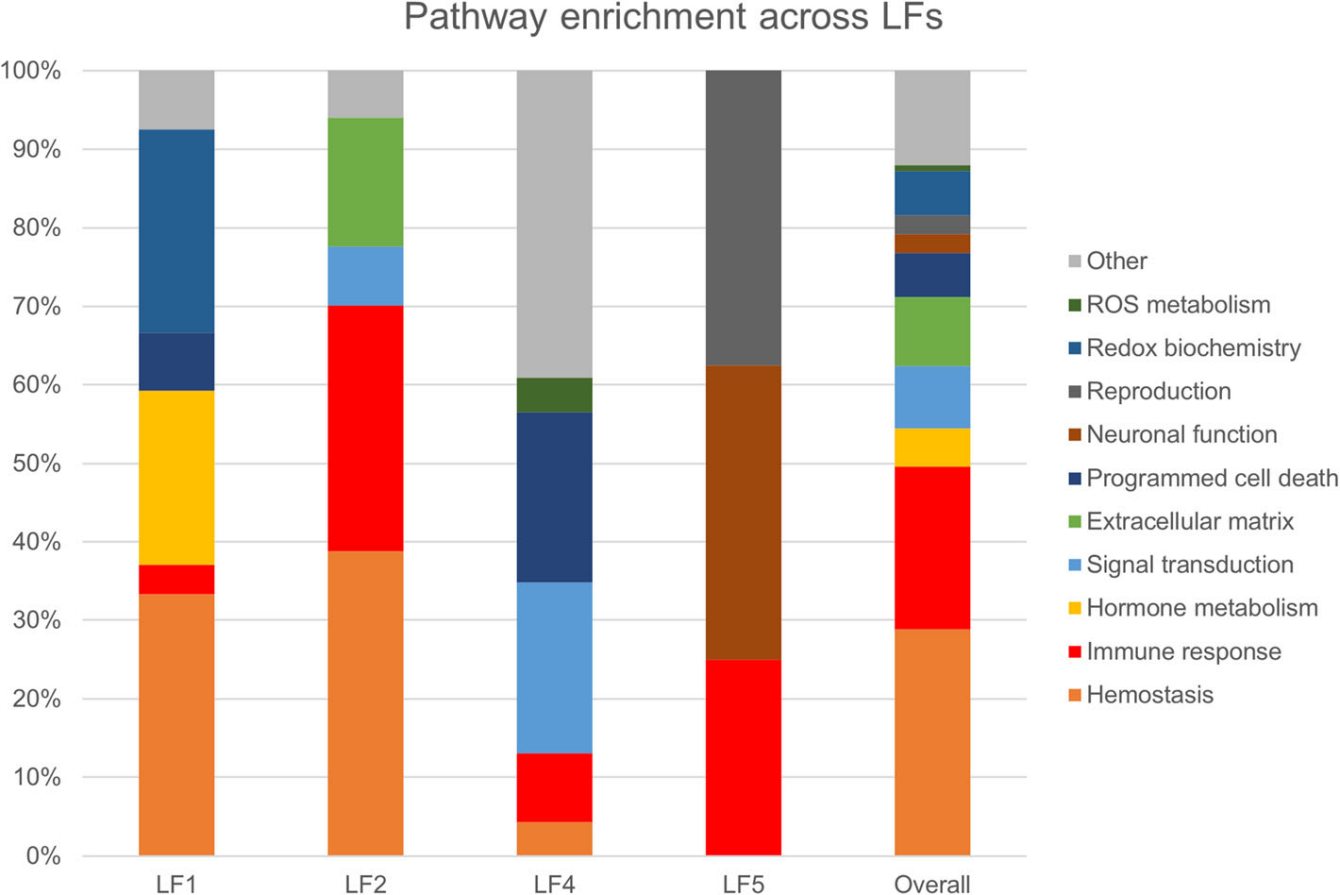
Pathway enrichment. Pathway enrichment analysis of identified proteins across LFs and overall. The number of over-represented categories within each LF (expressed as a percentage) as well as across all LFs is represented. NB: the low number of analytes associated with LF3 did not allow for an enrichment analysis

Using the Reactome database and coarse-grain ontological categories (See the “Methods” section and Additional file 3, Table S1), we investigated which biological pathways were over-represented within each LF for the proteomic modality. Other modalities were not analyzed in this fashion since they were selected a priori to represent distinct metabolic pathways (one-carbon metabolism and inflammatory markers) or did not contain enough molecules to conduct pathway analysis. Lipids were also excluded from this analysis since our quantification method did not allow to dissociate between different isoforms of compounds with the same chemical formula. This approach revealed an overrepresentation of the hemostasis (28.8%), immune response (20.8%), and extracellular matrix signaling pathways (8.8%) (Fig. 6).

## Discussion

Here, we applied a multi-layered integrative approach to disentangle sources of variance within a cohort of elderly participants with normal cognition, mild cognitive impairment, or mild AD dementia. We identified five major dimensions of heterogeneity that together comprehensively explained the variance within the cohort and were associated with core AD pathology. Further analysis revealed multiple interactions between single ‘omics modalities, distinct multi-omics molecular patterns differentially associated with amyloid aggregation, neurodegeneration, and tau hyperphosphorylation, and novel molecules associated with cognitive impairment. Specific signatures of four molecules improved the accuracy of both AD and cognitive decline prediction. Additionally, pathway enrichment showed over-representation of the hemostasis, immune response and extracellular matrix signaling pathways in association with AD.

### Single modality feature selection

We first used Elastic-net regression, to identify molecules associated with individual biomarkers of CSF AD pathology without considering any possible interactions between different ‘omics modalities. This approach identified several proteins (SPARC-related modular calcium-binding protein 1, brain acid soluble protein 1, neuromodulin, pyruvate kinase PKM, thymosin beta-10, 14-3-3 protein zeta/delta, and fructose-bisphosphate aldolase A) in strong accordance with recent studies of the AD CSF proteome [24, 65]. The zeta/delta isoform of protein 14-3-3 was associated with Aβ_1-42_, Tau, and P-Tau levels. This apoptosis inhibitor, one of the most abundant proteins in the brain, was previously found to exhibit altered levels in AD and modulate AD risk [66, 67]. We also identified associations of neurofilament medium polypeptide with Tau levels and of reelin with Aβ_1-42_ and Tau levels. Both these molecules have previously been associated with AD [68–70]. Regarding neuroinflammatory molecules, C-reactive protein and monocyte chemoattractant protein-1 have previously been associated with AD, albeit in plasma [71]. In addition, we have also previously shown that soluble intracellular cell adhesion molecule-1 in CSF is associated with AD [30]. At metabolite level, we identified 10 molecules in CSF associated with Tau and P-Tau, which differ from the blood biomarkers associated with AD identified in a recent study in a large sample [72]. Overall, our approach identified more molecules associated with AD pathology as compared to previous studies. A likely source of differences is the use of Elastic-Net regression in the current study which eliminates saturation of the regression and could therefore identify more associations. On the other hand, our approach did not identify any lipids or metabolites associated with Aβ_1-42_ levels. It is possible that it is not the individual levels of these molecules that are associated with Aβ_1-42_ levels, but rather lipidomic pathway alterations where associations of individual lipids are weak but the overall pathway in which they are embedded is strongly associated with amyloid pathology.

### Heterogeneity within the cohort

An important strength of our study is to consider all interactions between multiple biological levels and their associations with the heterogeneity within the cohort. This was achieved by training a MOFA model on the multi-omics dataset which has the advantage of not giving any additional analysis weight to the established CSF biomarkers of core AD pathology while also reducing the complexity of the data to better depict the sources of variation. This revealed proteomic measures and CSF core AD biomarkers as the main contributors to the variance with both having very similar contributions to variance, albeit from 768 proteins versus 3 AD biomarkers. The biomarker contribution was expected as our sample contains a large proportion of participants with AD, each displaying CSF AD biomarkers significantly different from subjects without AD. The large contribution of proteomics to variance could derive, at least in part, from the fact that protein expression levels reflect the effects of different environments, life style, health conditions, and genetic backgrounds; all factors potentially affecting protein expression and regulation [73]. Nonetheless, MOFA analysis identified 21 proteins with previously reported association to AD, suggesting, along with the associations with AD biomarkers observed here, that protein contribution to variance is linked to AD pathology. These findings further show that the MOFA approach can accurately disentangle the inter-individual heterogeneity driven by AD pathology and differentiate between individual (i.e., not repeated in the dataset) and cohort heterogeneity (i.e., underlying changes in many participants). Conversely, the metabolomic dataset was only responsible for a small amount of the cohort heterogeneity (3.7%), a possible explanation being that it represents individual heterogeneity for the most part caused by the environment, disease processes, or nutritional habits. This low contribution of metabolomics to variance could also result from the lower dimensionality of the metabolomics dataset as molecules within had lower concentrations compared to molecules in the other modalities. Yet, despite this low level of variance, our model was able to correctly retrieve metabolites previously reported in association with AD, underlining the sensitivity of the model. This is further supported by the ability of our approach to determine a four-molecule signature that improves the prediction of AD pathology.

### Associations between LF and specific aspects of AD pathology

Our analysis revealed that LFs 1–3 were primarily associated with neuronal injury while LF4 and LF5 were mainly associated with amyloid pathology. In addition, both Tau and P-Tau were negatively associated with LF3, while Aβ_1-42_ presented a positive, albeit weaker, association with this same LF. Conversely, Tau and P-Tau were positively while Aβ_1-42_ was strongly negatively associated with LF4. Whether the LF3 and LF4, that show congruent relationships with amyloid aggregation, tau pathology and neurodegeneration, may be particularly relevant for AD remains to be confirmed. The finding that the zeta/delta isoform of protein 14-3-3, selected in LF4, is associated with all AD CSF biomarkers along with its contribution to predictive models of cerebral AD is in line with the hypothesis that these LFs are involved in AD pathology.

### Interactions between LFs and ‘omics modalities

Besides the identification of molecular profiles and metabolic pathways alterations associated with AD, our approach offers the unique ability to disentangle how components of individual LFs interact with each other to explain variance within the cohort. This not only reveals specific interactions between subsets of molecules and particular metabolic pathways but also offers a unique view into how multiple biological levels interact in the context of AD pathology and how they are related to specific aspects of the pathology. In addition, this approach has the advantage of not being biased towards any known biological alteration of AD pathology or giving any particular weight to a specific molecule or metabolic pathway. In the context of AD, this could lead to the identification of pathways and alterations not directly related to the core features of AD pathology, better reflecting the heterogeneity of the disease. The presence of clusters within each LF also suggests specific groups of molecules interacting with each other across LFs. A more comprehensive analysis of the role of these clusters of analytes may be addressed in future studies.

### Novel associations uncovered by the MOFA model

The MOFA model uncovered additional relationships not revealed by other analysis exploration paradigms since it does not only consider molecules from one modality but the whole dataset from different ‘omics. These additional findings may result from the downstream effects of these molecules or from interactions with other modalities. These include the association of total cysteine with CSF Aβ_1-42_, Tau, and P-Tau or the association of kininogen-1 (KNG1) with Tau and P-Tau. While cysteine was previously linked with AD [60], KNG-1 has been associated with other neurodegenerative disorders [74], but its association with AD, and in particular tau pathology and neuronal injury, is novel. The MOFA model also identified molecules previously associated with cognitive impairment, such as dynein light-chain 2, cytoplasmic (DYL2), and neurexophilin-4 (NXPH4). Both were associated with LF1 and with cognitive impairment. DYL2 is thought to regulate dynein function [75] and maintain cytoskeletal structure, therefore regulating synaptic function [76]. NXPH4 structurally resembles neurexophilin-1, an α-neurexin ligand, which promotes adhesion between dendrites and axons and modulates specific cerebellar synapses and motor functions [77]. Altered levels of these proteins may therefore be associated with neurodegeneration processes and related to cognitive impairment in AD. Another novel analyte we identified is the cholesteryl ester SE 27:1 16:0. While links between phosphatidylcholine metabolism and AD in general [78] and PC 32:0 in particular [64] have been previously reported, to our knowledge, cholesteryl esters have not previously been associated with AD pathology. In our MOFA model, this cholesteryl ester was strongly correlated to LF4, suggesting a role in amyloid pathology. These molecules were also associated with cognitive performance as measured by MMSE. Together, these results demonstrate the capacity of integrative multi-omics to provide additional insights into the relationship of molecular alterations with specific aspects of the AD pathology.

### Prediction of AD pathology and cognitive decline using MOFA-selected molecules

Molecular signatures associated with AD or predictive of cognitive decline were derived from our model. Both signatures contain four molecules each, taken from multiple biological levels and significantly improved prediction performance when added to reference models. The four molecules from the combination related to AD pathology have each been associated with AD previously [38, 42, 49, 54]. From the molecule combination that improved prediction of cognitive decline, three molecules have been linked to cognitive decline in previous reports [79–81], while one molecule, cholesteryl ester 27:1 16:0 was not. Both signatures also share two common molecules, protein 14-3-3 zeta/delta and clusterin, suggesting these belong to common biological pathways both associated with AD and relevant for cognitive decline. Cholesteryl ester 27:1 16:0 and monocyte chemoattractant protein-1 may indicate pathway alterations without a strong and direct link to core AD pathology but having impact on the rapidity of cognitive decline. These predictive models also demonstrate the ability of this approach to identify biomarker candidates for both AD pathology and cognitive decline. Additional investigation and validation in independent cohorts is required before possible clinical use.

### Infer pathway relationships with AD pathology

One important strength of the MOFA approach is that it enables addressing the relationship between multiple biological pathways and associate them with sources of variance (i.e., LFs). Using over-representation of metabolic pathways, we were able to show that individual LFs, and the main related pathological aspects of AD (i.e., amyloid aggregation, neurodegeneration and tau pathology) are associated with distinct pathways. Hemostasis and immune response were the most over-represented. Only the immune response was associated with all LFs in which individual pathways could be identified. LF1 and LF2 presented a significant enrichment in biomolecules implicated in hemostasis, suggesting an association between this pathway and neuronal injury, and tau pathology. While an association between hemostasis and amyloid pathology pathway was previously described [82], in particular related to expression of amyloid precursor protein and release of Aβ [83], there have also been recent reports of an association between Tau and hemostasis [84]. Molecules involved in the extracellular matrix were significantly enriched in LF2, also suggesting an association with tau-related pathology, in line with previous reports [85]. However, this pathway was not detected within LF1 or other LFs. We therefore hypothesize that the molecules involved are those presenting a specific pattern of association with LF2, such as PC 32:0, PC 34:1, LPA 18:3, and TAG 54:3. Neuronal function was confined to associations with LF5, suggesting little variation and differences in signal transmission and synaptic function across the cohort since this LF only explained 8% of the variance. Nonetheless, this result suggests an association with amyloid pathology, which is in accordance with previous findings of amyloid being released in an activity-dependent fashion from neurons and modulating synaptic function and plasticity [86, 87]. Overall, the enriched metabolic pathways suggest that AD pathology affects not only pathways related to neuronal biological systems but is linked to a broader spectrum of metabolic dysfunctions.

### Limitations

A limitation of this study is the different amounts of analytes measured by individual quantification method (i.e., > 500 proteins measured vs. < 100 metabolites/lipids for example) resulting from methodological differences. This approach prevents measuring the relative importance of the analytes or their combinations but allows the identification of altered pathways or molecular signatures from different modalities. Furthermore, since the data entered in the MOFA analyses did not include information regarding clinical stages, stage-specific alterations have not been addressed. Also, the inclusion of some targeted analysis results in the multi-omics models may be considered as a limitation. While the proteomic and lipidomic dataset are hypothesis-free measurements and the study could be limited to this data, we chose to include further available modalities. In particular, we considered neuroinflammation and one-carbon metabolism given their previously reported associations with AD and relevance for brain metabolism. However, the replication of these and other previously reported associations in our MOFA model along with the ability of the MOFA selected marker combinations to improve prediction of AD and of cognitive decline support the validity of the new findings revealed in the present study.

## Conclusions

Here, applying integrative multi-omics in AD, we have identified five axes of variation within a cohort of individuals with normal cognition or with cognitive impairment. These five LFs were associated with different aspects of the core AD pathology. We confirmed several previously reported associations with AD and identified new molecular patterns interrelated within each LF. Additionally, we identified molecular biomarker signatures improving the diagnosis of AD pathology and the prediction of future cognitive decline. Furthermore, using pathway enrichment analysis, we have revealed metabolic pathways represented within single LFs and explored specific relationships with markers of amyloid pathology, neuronal injury, and tau hyperphosphorylation. These findings demonstrate the added value of integrative multi-omics analysis to uncover interrelated pathway alterations in AD and its ability to identify biomarker combinations that may be used in clinical practice. This is relevant for the development of both personalized diagnosis and tailored therapeutic interventions in AD.

## Supplementary Information

**Additional file 1.** Supplementary methods used in this study, including additional details on omics techniques used for quantification, validation of the MOFA model and association with clinical measurements.

**Additional file 2: **Additional Figures, including a correlation matrix analysis of latent factors (**Figure S1**) and prediction of CSF AD biomarkers using the trained MOFA model (**Figure S2**).

**Additional file 3: **Additional Tables, including coarse-grain categories used for pathway enrichment (**Table S1**), relationship between selected CSF molecules and latent factors (**Table S2**), correlations between selected proteins and lipids with CDR-SoB and MMSE scores (**Tables S3**) and CSF proteins presenting an association with cognitive impairment (**Table S4**).

## Data Availability

Proteomics data used for this study was previously deposited to the ProteomeXchange Consortium (http://proteomecentral.proteomexchange.org) via the PRIDE partner repository and is available with the identifier PXD009589. The other datasets used and/or analyzed during the current study are available from the corresponding author on reasonable request.

## Abbreviations

Aβ_1-42_: CSF beta-amyloid 1-42
AD: Alzheimer’s disease
AIC: Akaike information criterion
AUC: Area under the curve
CDR: Clinical Dementia Rating
CDR-SoB: CDR sum of boxes
CSF: Cerebrospinal fluid
^1^H NMR: Proton nuclear magnetic resonance
LF: Latent factor
MCI: Mild cognitive impairment
MMSE: Mini mental state examination
MOFA: Multi-omics factor analysis
MS: Mass spectrometry
P-Tau: Tau phosphorylated at threonine 181
Tau: Total-tau
