# Supplementary material for: An integrative multi-omics approach reveals new central nervous system pathway alterations in Alzheimer’s disease
Source: Alzheimers Res Ther. 2021 Apr 1;13:71. doi: 10.1186/s13195-021-00814-7 (PMC8015070; doi:10.1186/s13195-021-00814-7)
Supplement: Supplementary file 1 — Additional file 1. Supplementary methods used in this study, including additional details on omics techniques used for quantification, validation of the MOFA model and association with clinical measurements. [file 13195_2021_814_MOESM1_ESM.docx]

**Additional File 1**

**Supplementary Methods:**

**Multi-omics Factor Analysis (MOFA):**

The MOFA method is an hypothesis free Bayesian approach (*i.e.,* which does not depend on the assumptions of frequentist statistics such as large amount of data or Gaussian distribution). MOFA is best viewed as a generalization of principal component analysis. It can infer a low-dimensional data representation from multiple measurements at different multi-omics levels in terms of latent factors that capture major sources of variation within the data. Furthermore, MOFA identifies to what extent factors are associated with each data modality. MOFA also determines the loadings of individual analytes, providing the mapping between the high-dimensional space (the datasets) and the low-dimensional space (the factors). A score for each analyte on each factor is determined, such that analytes with no association with the factor are expected to have values close to zero, whereas those with strong association with the factor are expected to have large values. Absolute loadings derived from the MOFA model are relative within any given LF. This approach also has the advantage of being able to deal with missing values and does not require any normalization, centering or scaling of variables before analysis.

**Validation of trained MOFA model:**

***Correlation of latent factors:*** A correlation matrix for all latent factors was obtained using Pearson’s correlation coefficient. This was used to determine independence of latent factors (**Figure S1**).

***Prediction of CSF AD biomarkers:*** In order to assess if the trained MOFA model correctly represented variance in the cohort, we used it to predict another set of CSF AD biomarker values and compared these to the original values within the cohort. Prediction accuracy was evaluated using root mean squared error (*i.e.*, the square root of the mean squared error) for each individual biomarker as well as for the overall prediction (**Figure S2;** root mean squared error of 0.117 for Aβ_1-42_, 0.221 for Tau, 0.151 for P-Tau and 0.221 overall).

***Comparison with other approaches:*** We also compared the list of analytes identified by our trained MOFA model with the literature and found that 21 out of 36 proteins, a single lipid (PC 32:0), and all one-carbon metabolites and neuroinflammatory markers (**Table 6**) identified by our model have previously been associated with AD pathology, confirming the validity of the approach. Additionally, single-omics approaches on individual parts of this dataset, identified the same molecules associated with AD, confirming our results (1).

**Associations with clinical measures:**

***Correlation with CDR-SoB and MMSE scores:*** In order to evaluate the association of the analytes identified by the MOFA model with clinical scores, we investigated their associations with cognitive status (classified using CDR) by logistic binomial regression along with their correlations with both MMSE and CDR-SoB scores. Correlations were assessed with one-sided Spearman rho. Logistic binomial regressions were performed with the presence of cognitive impairment as dependent variable and all features identified by the MOFA model entered in the model. Twenty-eight out of thirty-seven proteins were correlated with MMSE score and a different subset of thirteen correlated with CDR-SoB score (**Table S3**). Fourteen out of thirty-seven proteins were further associated with the presence of cognitive impairment at baseline in regression models (**Table S4**). None of the five identified lipids were associated with cognitive impairment at baseline, but three of them were correlated with both CDR-SoB and MMSE scores (**Table S3**).

**References:**

1. Dayon L, Guiraud SP, Corthesy J, Da Silva L, Migliavacca E, Tautvydaite D et al. One-carbon metabolism, cognitive impairment and CSF measures of Alzheimer pathology: homocysteine and beyond. Alzheimer’s research & therapy 2017; 9(1):43.
