# Supplementary material for: An integrative multi-omics approach reveals new central nervous system pathway alterations in Alzheimer’s disease
Source: Alzheimers Res Ther. 2021 Apr 1;13:71. doi: 10.1186/s13195-021-00814-7 (PMC8015070; doi:10.1186/s13195-021-00814-7)
Supplement: Supplementary file 2 — Additional file 2: Additional Figures, including a correlation matrix analysis of latent factors (Figure S1) and prediction of CSF AD biomarkers using the trained MOFA model (Figure S2). [file 13195_2021_814_MOESM2_ESM.docx]

**Additional File 2**

**Supplementary Figures:**

**Figure S1:** Correlation matrix


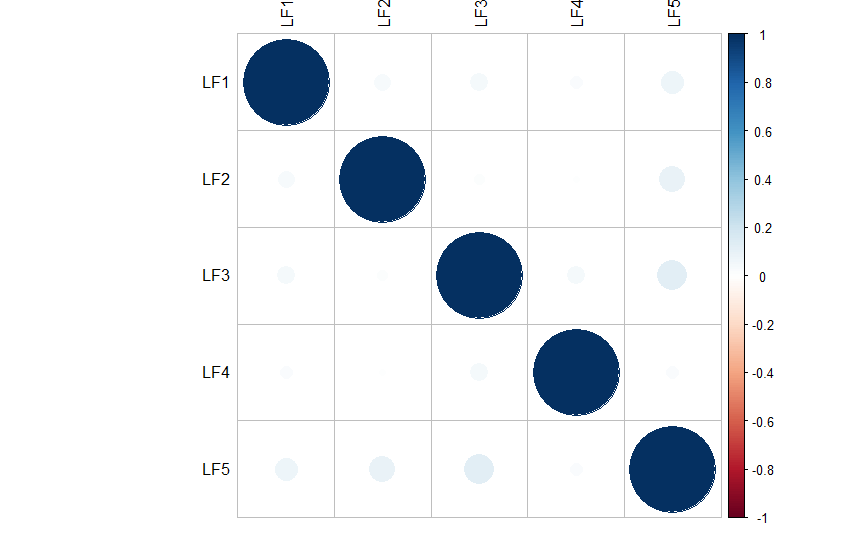


Pearson’s correlation matrix analysis of latent factors discovered by the trained MOFA model.

**Figure S2:** Prediction of AD biomarkers


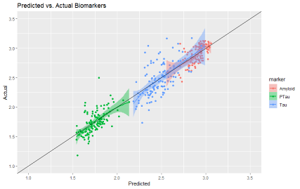


Prediction of CSF AD biomarkers using the trained MOFA model for Aβ_1-42_ (red), Tau (blue) and P-Tau (green). Actual biomarker values are projected against predicted values. The black line denotes a line of slope 1. Shaded area represents smoothed means.
