# Supplementary material for: An integrative multi-omics approach reveals new central nervous system pathway alterations in Alzheimer’s disease
Source: Alzheimers Res Ther. 2021 Apr 1;13:71. doi: 10.1186/s13195-021-00814-7 (PMC8015070; doi:10.1186/s13195-021-00814-7)
Supplement: Supplementary file 3 — Additional file 3: Additional Tables, including coarse-grain categories used for pathway enrichment (Table S1), relationship between selected CSF molecules and latent factors (Table S2), correlations between selected proteins and lipids with CDR-SoB and MMSE scores (Tables S3) and CSF proteins presenting an association with cognitive impairment (Table S4). [file 13195_2021_814_MOESM3_ESM.docx]

**Additional File 3**

**Supplementary Tables:**

**Table S1:** Ontological categories

| **Latent Factor 1** |  |
| --- | --- |
| **Pathway identifier** | **Pathway name** |
|  |  |
| **Redox biochemistry** |  |
| *R-HSA-5661231* | Metallothioneins bind metals |
| *R-HSA-5660526* | Response to metal ions |
| *R-HSA-8953897* | Cellular responses to external stimuli |
|  |  |
| **Hemostasis** |  |
| *R-HSA-114608* | Platelet degranulation |
| *R-HSA-76005* | Response to elevated platelet cytosolic Ca2+ |
| *R-HSA-76002* | Platelet activation, signaling and aggregation |
|  |  |
| **Immune response** |  |
| *R-HSA-166665* | Terminal pathway of complement |
|  |  |
| **Hormone metabolism** |  |
| *R-HSA-209952* | Peptide hormone biosynthesis |
| *R-HSA-400511* | Synthesis, secretion, and inactivation of Glucose-dependent Insulinotropic Polypeptide (GIP) |
| *R-HSA-381771* | Synthesis, secretion, and inactivation of Glucagon-like Peptide-1 (GLP-1) |
| *R-HSA-422085* | Synthesis, secretion, and deacylation of Ghrelin |
| *R-HSA-400508* | Incretin synthesis, secretion, and inactivation |
| *R-HSA-264876* | Insulin processing |
|  |  |
| **Programmed cell death** |  |
| *R-HSA-139910* | Activation of BMF and translocation to mitochondria |
| *R-HSA-114452* | Activation of BH3-only proteins |
|  |  |
| **Other** |  |
| *R-HSA-186763* | Downstream signal transduction |
| *R-HSA-2129379* | Molecules associated with elastic fibres |
|  |  |
| **Latent Factor 2** |  |
| **Pathway identifier** | **Pathway name** |
|  |  |
| **Hemostasis** |  |
| *R-HSA-114608* | Platelet degranulation |
| *R-HSA-76005* | Response to elevated platelet cytosolic Ca2+ |
| *R-HSA-76002* | Platelet activation, signaling and aggregation |
| *R-HSA-109582* | Hemostasis |
| *R-HSA-140877* | Formation of Fibrin Clot (Clotting Cascade) |
| *R-HSA-75205* | Dissolution of Fibrin Clot |
| *R-HSA-140837* | Intrinsic Pathway of Fibrin Clot Formation |
| *R-HSA-140875* | Common Pathway of Fibrin Clot Formation |
| *R-HSA-76009* | Platelet Aggregation (Plug Formation) |
|  |  |
| **Signal transduction** |  |
| *R-HSA-381426* | Regulation of Insulin-like Growth Factor (IGF) transport and uptake by Insulin-like Growth Factor Binding Proteins (IGFBPs) |
| *R-HSA-5674135* | MAP2K and MAPK activation |
|  |  |
| **Immune response** |  |
| *R-HSA-166665* | Terminal pathway of complement |
| *R-HSA-977606* | Regulation of Complement cascade |
| *R-HSA-166658* | Complement cascade |
| *R-HSA-168249* | Innate Immune System |
| *R-HSA-5686938* | Regulation of TLR by endogenous ligand |
| *R-HSA-168256* | Immune System |
|  |  |
| **Extracellular matrix** |  |
| *R-HSA-216083* | Integrin cell surface interactions |
| *R-HSA-1474244* | Extracellular matrix organization |
| *R-HSA-372708* | p130Cas linkage to MAPK signaling for integrins |
| *R-HSA-354194* | GRB2:SOS provides linkage to MAPK signaling for Integrins |
| *R-HSA-3000170* | Syndecan interactions |
| *R-HSA-354192* | Integrin signaling |
| *R-HSA-1592389* | Activation of Matrix Metalloproteinases |
| *R-HSA-2129379* | Molecules associated with elastic fibres |
|  |  |
| **Other** |  |
| *R-HSA-8957275* | Post-translational protein phosphorylation |
| *R-HSA-6802948* | Signaling by high-kinase activity BRAF mutants |
|  |  |
| **Latent Factor 4** |  |
| **Pathway identifier** | **Pathway name** |
|  |  |
| **Signal transduction** |  |
| *R-HSA-9013700* | NOTCH4 Activation and Transmission of Signal to the Nucleus |
| *R-HSA-3769402* | Deactivation of the beta-catenin transactivating complex |
| *R-HSA-9604323* | Negative regulation of NOTCH4 signaling |
| *R-HSA-5625740* | RHO GTPases activate PKNs |
| *R-HSA-9013694* | Signaling by NOTCH4 |
|  |  |
| **Hemostasis** |  |
| ***R-HSA-430116*** | GP1b-IX-V activation signalling |
|  |  |
| **Programmed cell death** |  |
| *R-HSA-111447* | Activation of BAD and translocation to mitochondria |
| *R-HSA-114452* | Activation of BH3-only proteins |
| *R-HSA-109606* | Intrinsic Pathway for Apoptosis |
| *R-HSA-109581* | Apoptosis |
| *R-HSA-5357801* | Programmed Cell Death |
|  |  |
| **Immune response** |  |
| *R-HSA-392517* | Rap1 signalling |
| *R-HSA-512988* | Interleukin-3, Interleukin-5 and GM-CSF signaling |
|  |  |
| **ROS metabolism** |  |
| *R-HSA-3299685* | Detoxification of Reactive Oxygen Species |
|  |  |
| **Other** |  |
| *R-HSA-9614399* | Regulation of localization of FOXO transcription factors |
| *R-HSA-75035* | Chk1/Chk2(Cds1) mediated inactivation of Cyclin B:Cdk1 complex |
| *R-HSA-450604* | KSRP (KHSRP) binds and destabilizes mRNA |
| *R-HSA-9614085* | FOXO-mediated transcription |
| *R-HSA-1445148* | Translocation of SLC2A4 (GLUT4) to the plasma membrane |
| *R-HSA-69473* | G2/M DNA damage checkpoint |
| *R-HSA-5628897* | TP53 Regulates Metabolic Genes |
| *R-HSA-450531* | Regulation of mRNA stability by proteins that bind AU-rich elements |
| *R-HSA-69481* | G2/M Checkpoints |
|  |  |
| **Latent Factor 5** |  |
| **Pathway identifier** | **Pathway name** |
|  |  |
| **Reproduction** |  |
| *R-HSA-2534343* | Interaction with Cumulus Cells And The Zona Pellucida |
| *R-HSA-1187000* | Fertilization |
| *R-HSA-1474165* | Reproduction |
|  |  |
| **Neuronal function** |  |
| *R-HSA-388844* | Receptor-type tyrosine-protein phosphatases |
| *R-HSA-6794362* | Protein-protein interactions at synapses |
| *R-HSA-112316* | Neuronal System |
|  |  |
| **Immune response** |  |
| *R-HSA-6803157* | Antimicrobial peptides |
| *R-HSA-168249* | Innate Immune System |

Coarse-grain categories used for pathway enrichment within each latent factor. Pathway identifiers are extracted from the Reactome database.

**Table S2**: Correlations of MOFA selected analytes with CSF AD biomarkers

|  | LF | Aβ_1-42_ | Tau | P-Tau |  | LFs | Aβ_1-42_ | Tau | P-Tau |
| --- | --- | --- | --- | --- | --- | --- | --- | --- | --- |
| ***Proteomics*** |  |  |  |  | ***Neuroinflammation*** |  |  |  |  |
| NRN1 | 1 | **0,252**** | **0,473***** | **0,501***** | sVCAM-1 | 1 | 0.045 | **0.577***** | **0.568***** |
| SMS | 1 | **0,403***** | **0,367***** | **0,467***** | IL-15 | 1 | 0.022 | **0.676***** | **0.664***** |
| NXPH4 | 1 | **0,280**** | **0,383***** | **0,413***** | sICAM-1 | 1 | -0.078 | **0.535***** | **0.513***** |
| LTBP1 | 1 | 0,125 | **0,514***** | **0,534***** | SAA | 2 | 0.091 | 0.097 | 0.083 |
| CLUS | 1 | 0,143 | **0,448***** | **0,467***** | PIGF_1R | 2,3,5 | -0.188 | 0.120 | 0.094 |
| NPDC1 | 1 | **0,257**** | **0,503***** | **0,561***** | IL-16 | 4 | **0.235*** | **0.206*** | **0.221*** |
| PNOC | 1 | **0,332***** | **0,417***** | **0,467***** | MCP-1 | 5 | **-0.292**** | **0.419***** | **0.346***** |
| DYL2 | 1 | 0,128 | **0,604***** | **0,633***** |  |  |  |  |  |
| PDGFB | 1 | 0,183 | **0,524***** | **0,559***** | ***One-carbon metabolism*** |  |  |  |  |
| SAP3 | 1 | **0,229*** | **0,480***** | **0,528***** | MTHF | 1 | 0.013 | **-0.384***** | **-0.405***** |
| MT1E | 1 | **0,203*** | **0,507***** | **0,539***** | SAH | 1,3 | 0.086 | **0.365***** | **0.371***** |
| PCSK1 | 1 | **0,236*** | **0,433***** | **0,473***** | CYST | 2,4,5 | -0.036 | 0.115 | 0.091 |
| TAGL | 1 | 0,008 | **0,457***** | **0,461***** |  |  |  |  |  |
| MT3 | 1 | **0,207*** | **0,500***** | **0,521***** | ***Metabolomics*** |  |  |  |  |
| LY6H | 1 | **0,275**** | **0,503***** | **0,580***** | Glycoproteins | 1,4 | **0.219*** | **0.572***** | **0.593***** |
| SAMP | 2 | -0,074 | 0,02 | -0,063 | Alanine | 2 | -0.068 | -0.020 | -0.048 |
| VTNC | 2 | -0,004 | 0,097 | 0,099 | Valine | 2 | -0.040 | -0.090 | -0.092 |
| KNG1 | 2 | -0,096 | 0,043 | 0,021 | Inositol | 3 | 0.113 | -0.179* | -0.154 |
| FETUA | 2 | 0,007 | -0,018 | -0,021 | Formic Acid | 5 | 0.007 | 0.097 | 0.046 |
| HELZ | 2 | -0,055 | 0,011 | -0,022 | S69 | 5 | 0.135 | **0.261**** | **0.287**** |
| PLMN | 2 | -0,114 | 0,064 | 0,035 | Acetoacetic Acid | 5 | -0.086 | 0.090 | 0.018 |
| PGRP2 | 2 | -0,173 | 0,052 | 0,038 |  |  |  |  |  |
| AFAM | 2 | -0,146 | -0,116 | -0,113 | ***Lipidomics*** |  |  |  |  |
| ITIH1 | 2 | -0,113 | 0,06 | 0,02 | PC 32:0 | 1,3 | 0.153 | **0.608***** | **0.598***** |
| CO8B | 2 | -0,14 | 0,093 | 0,06 | SE 27:1 18:2 | 2,4 | -0.165 | -0.070 | -0.112 |
| FIBA | 2 | -0,033 | 0,183 | 0,171 | SE 27:1 20:4 | 4 | -0.065 | 0.148 | 0.125 |
| CO6 | 2 | -0,107 | 0,065 | 0,055 | SE 27:1 16:0 | 4 | -0.096 | **0.357***** | **0.276**** |
| ITIH4 | 2 | -0,04 | 0,171 | 0,156 | LPG 20:1 | 5 | -0.083 | -0.077 | -0.055 |
| EPDR1 | 3 | **0,281**** | 0,136 | 0,191 |  |  |  |  |  |
| SIAE | 3 | 0,096 | -0,146 | -0,087 |  |  |  |  |  |
| X1433Z | 4 | -0,2 | 0,604 | 0,592 |  |  |  |  |  |
| S10A6 | 4,5 | 0,023 | 0,3 | 0,278 |  |  |  |  |  |
| PRDX6 | 4 | 0,078 | 0,29 | 0,285 |  |  |  |  |  |
| VTM2A | 5 | **0,253**** | 0,564 | 0,625 |  |  |  |  |  |
| CMGA | 5 | **0,280**** | 0,52 | 0,583 |  |  |  |  |  |
| ZP2 | 5 | **0,252**** | 0,294 | 0,286 |  |  |  |  |  |
| SLIK1 | 5 | **0,301***** | 0,493 | 0,558 |  |  |  |  |  |

CSF analytes identified by the MOFA model and their correlations (two-tailed rho) with CSF AD biomarkers. Analytes are labelled according to **Table 6**. The LFs in which each analyte was identified is shown. * p-value < 0.05; ** p-value < 0.01; *** p-value < 0.001.

**Table S3:** Correlations with CDRSoB and MMSE scores

| PROTEIN | CDR-SoB | MMSE |  | LIPID | CDR-Sob | MMSE |
| --- | --- | --- | --- | --- | --- | --- |
| NXPH4 | 0.067 | -0.209^*^ |  | **PC 32:0** | 0.047 | -0.100 |
| LTBP1 | 0.087 | -0.205^*^ |  | **SE 27:1 16:0** | 0.246^**^ | -0.244^**^ |
| CLUS | 0.127 | -0.253^**^ |  | **SE27:1 20:4** | 0.323^**^ | -0.330^**^ |
| NPDC1 | 0.051 | -0.160^*^ |  | **SE27:1 18:2** | 0.345^**^ | -0.383^**^ |
| DYL2 | 0.096 | -0.203^*^ |  | **LPG 20:1** | 0.077 | -0.065 |
| PDGFB | 0.100 | -0.204^*^ |  |  |  |  |
| SAP3 | 0.085 | -0.209^*^ |  |  |  |  |
| MT1E | 0.122 | -0.213^*^ |  |  |  |  |
| PCSK1 | 0.050 | -0.175^*^ |  |  |  |  |
| TAGL | 0.159^*^ | -0.209^*^ |  |  |  |  |
| MT3 | 0.049 | -0.187^*^ |  |  |  |  |
| SAMP | 0.142 | -0.237^**^ |  |  |  |  |
| VTNC | 0.191^*^ | -0.200^*^ |  |  |  |  |
| KNG1 | 0.233^**^ | -0.203^*^ |  |  |  |  |
| HELZ | 0.154 | -0.163^*^ |  |  |  |  |
| PLMN | 0.234^**^ | -0.255^**^ |  |  |  |  |
| PGRP2 | 0.249^**^ | -0.256^**^ |  |  |  |  |
| AFAM | 0.171^*^ | -0.159^*^ |  |  |  |  |
| ITIH1 | 0.224^**^ | -0.174^*^ |  |  |  |  |
| CO8B | 0.228^**^ | -0.208^*^ |  |  |  |  |
| FIBA | 0.278^**^ | -0.201^*^ |  |  |  |  |
| CO6 | 0.225^**^ | -0.190^*^ |  |  |  |  |
| ITIH4 | 0.250^**^ | -0.217^*^ |  |  |  |  |
| EPDR1 | 0.024 | -0.199^*^ |  |  |  |  |
| SIAE | 0.090 | -0.248^**^ |  |  |  |  |
| X1433Z | 0.158^*^ | -0.141 |  |  |  |  |
| S10A6 | 0.145 | -0.229^**^ |  |  |  |  |
| PRDX6 | 0.173^*^ | -0.243^**^ |  |  |  |  |
| ZP2 | 0.063 | -0.209^*^ |  |  |  |  |

Spearman’s rho correlations between selected proteins (left) and lipids (right) with CDR-SoB and MMSE scores. * p-value < 0.05; ** p-value < 0.01.

**Table S4:** Associations with cognitive impairment

| UniProt entry name | B. coeff | Sig. |
| --- | --- | --- |
| SMS | -13.741 | 0.010 |
| NXPH4 | 13.156 | 0.006 |
| LTBP1 | -11.981 | 0.014 |
| CLUS | -22.558 | 0.013 |
| NPDC1 | 23.291 | 0.028 |
| DYL2 | 31.983 | 0.003 |
| TAGL | -7.578 | 0.044 |
| FETUA | -8.614 | 0.044 |
| PGRP2 | 23.574 | 0.004 |
| AFAM | 10.723 | 0.020 |
| FIBA | 15.355 | 0.010 |
| CO6 | -18.412 | 0.014 |
| ITIH4 | -26.455 | 0.010 |
| X1433Z | 18.030 | 0.025 |

CSF proteins identified by the MOFA model presenting an association with cognitive impairment at baseline. β-coefficients and significance obtained by binary logistic regression models are shown.
